# Axial ablation versus terminal interruption of the reflux source (AAVTIRS): a randomised controlled trial

**DOI:** 10.1186/s13063-022-06440-4

**Published:** 2022-06-10

**Authors:** C. R. Keohane, D. Westby, M. Twyford, T. Ahern, W. Tawfick, S. R. Walsh

**Affiliations:** 1 University Hospital Galway, Galway, Ireland; 2 Roscommon University Hospital, Roscommon, Ireland; 3 National University of Ireland Galway, Galway, Ireland; 4 Lambe Institute for Translational Research, National University of Ireland Galway, Newcastle Rd, Galway, Ireland

## Abstract

**Background:**

Treatment of superficial venous reflux has been shown to improve ulcer healing time and reduce the risk of ulcer recurrence. Terminal ablation of the reflux source (TIRS) is an alternative to formal endovenous ablation or surgery which can be performed by injecting sclerosant foam into the peri-ulcer plexus of the veins. TIRS has been shown to be successful and in our experience is the option preferred by many patients, when offered as an alternative to axial ablation (AA).

**Aim:**

To determine if the proportion of ulcers healed within 6 months of endovenous treatment differs between patients undergoing AA of varicose veins or TIRS by peri-ulcer foam sclerotherapy.

**Methods:**

AAVTIRS is an assessor-blinded randomised controlled trial. Patients will be recruited from a dedicated ulcer clinic in Roscommon University Hospital and from the vascular surgical clinics in University Hospital Galway. All patients attending the ulcer clinic will be screened for eligibility.

**Randomisation:**

Random computer-generated sequence is stratified by ulcer size. Allocation will be concealed using sealed opaque envelopes.

**Blinding:**

Assessors reviewing wounds at follow -p visits will be blinded to patient allocation.

**Primary endpoint:**

The proportion of ulcers healed within 6 months of enrolment.

**Discussion:**

This will be the first time that TIRS has been evaluated with a properly powered randomised trial in the setting of venous ulcer management. Streamlining the management of venous ulcers has broad health economic benefits. If it is found that TIRS is superior or non-inferior to AA, then a less expensive, less invasive injection can be offered as an alternative to AA in an attempt to encourage the healing of venous ulcers. If AA is found to be superior to TIRS, then this would suggest that all patients undergoing ablation in the management of venous ulcers should have their superficial reflux fully treated, building on the evidence of the EVRA trial.

**Trial registration:**

ClinicalTrials.gov NCT04484168. Registered on 23 July 2020

**Supplementary Information:**

The online version contains supplementary material available at 10.1186/s13063-022-06440-4.

## Administrative information

**Table:** 

**Trial synopsis**	
**Title**	Axial ablation versus terminal interruption of the Reflux Source in venous ulcer management: a randomised controlled trial in venous ulcer management
**Public title**	AAVTIRS trial
**Medical condition under investigation**	Lower limb venous ulcers
**Purpose of trial**	To evaluate whether there is a clinically significant difference between axial ablation of varicose veins and peri-ulcer terminal interruption of the reflux source in promoting healing of lower limb venous ulcers.
**Primary objective**	To determine if the proportion of ulcers healed within 6 months of endovenous treatment differs between patients undergoing axial ablation of varicose veins or terminal interruption of the reflux source by peri-ulcer foam sclerotherapy.
**Secondary objectives**	To determine whether there is a significant advantage to either treatment, in a reduction in ulcer size, encouraging wound regeneration, and venous disease severity or a significant difference in patient-assessed quality of life, between the two interventions
**Trial design**	Prospective single-centre assessor-blinded randomised controlled trial
**Primary endpoint**	Proportion of ulcers healed within 6 months of intervention
**Secondary endpoints**	Absolute and relative reduction in ulcer size Time to ulcer healing BWAT score progression VCSS progression over time Change in Charing Cross Venous Ulcer Questionnaire score from treatment to completion of follow-up
**Sample size**	320 individual ulcers
**Summary of eligibility**	Patients with a leg ulcer, attributable to venous insufficiency, without any contraindication to compression or either of the comparator treatment modalities will be included.
**Screening process**	All patients with a venous ulcer attending a Leg Ulcer Centre Ireland—a dedicated venous ulcer assessment clinic in Roscommon University Hospital will be considered for enrolment.
**Randomisation**	Affected legs will be randomised in a 1:1 ratio to axial ablation or terminal interruption of the reflux source.
**Treatment**	Patients will be treated on the same day as randomisation according to their assignment to either group
**Follow-up**	Patients will be followed monthly for 6 months.
**End-of-study**	The trial will end when all patients have been enrolled and completed both treatment and 6-month follow-up.
**Safety monitoring**	The principal investigator will be notified of any serious adverse events, and they will liaise with all investigators and the ethics committee.
**Criteria for stopping trial on safety grounds**	Unacceptable risk to participants due to unexpected serious adverse events Unacceptable rate of adverse events Interim demonstration of a significant difference in efficacy Interim demonstration of a significant difference in the rate of adverse or serious adverse events Intolerable discomfort during treatment or compression, or inability to adhere to the protocol for any other safety-related reason leading to insufficient compliance with randomisation
**Registration**	ClinicalTrials.gov registration NCT04484168. Registered on 23/7/2020
**Recruitment**	Commenced on 21 July 20—ongoing. Planned completion 1st quarter of 2022, last patient last visit 3rd quarter of 2022
**Recruiting country**	Ireland
**IPD sharing**	Not planned
**Funding**	No funding has been received for this research.
**Ethics**	Approved by the Galway University Hospitals Clinical Research Ethics Committee 18 June 2020, ref. no. C.A. 2416

## Background

Venous ulceration poses a massive burden on health services internationally [1–4]. In Ireland, the prevalence of leg ulcers is 1 per 800 people in the general population, rising to 1 per 100 over the age of 70 years [1]. Compression therapy has been shown to expedite the healing of venous ulcers [5, 6]. However, it is time-consuming for patients and practitioners and requires significant community support.

Superficial venous reflux is frequently present in patients with venous leg ulcers [7]. Surgical treatment of varicose veins, in combination with compression, has been shown to improve the rate of ulcer recurrence, but the ESCHAR trial did not show any benefit in ulcer healing from surgery [8, 9]. Therefore, many guidelines only recommend surgery as a means to reduce recurrence [10]. Numerous observational studies [11–14] and in particular the Early Venous Reflux Ablation (EVRA) trial have demonstrated that after endovenous treatment of reflux, ulcers do tend to heal faster, reducing ulcer persistence at 6 months [15]. This has important implications both for the patient in the psychosocial burden of venous ulceration [16] and for the economic burden of ulcer management on community, outpatient and inpatient services.

Endovenous management of reflux is achieved by ablation of the main superficial veins of the leg, or axial ablation (AA). If incompetent, the long or short saphenous veins, or their larger tributaries, contribute to venous ulceration by raising venous pressure, and therefore capillary hydrostatic pressure, leading to oedema, relative under-perfusion and hypoxia, and triggering an inflammatory cascade [17]. AA encourages healing by reducing venous reflux, decreasing venous pressure in distal veins, which improves the perfusion pressure at the capillary level. This improves oxygen and nutrient delivery to ulcerated areas, facilitating wound healing. Terminal interruption of the reflux source (TIRS) aims to target those veins in the distal leg which are directly responsible for transmitting the raised venous pressure to the ulcerated area and, by only treating these specific veins, achieve the necessary improvement in perfusion locally. While a review of the level 1 evidence has shown less anatomical success with ultrasound-guided foam sclerotherapy, clinical success was similar [18]. Foam sclerotherapy has been shown to be safe and effective in the management of venous ulcers [19, 20]. The ability to deliver foam via a small needle without anaesthetic makes foam sclerotherapy ideal for TIRS, and studies of perilesional sclerotherapy have found it to be safe and effective [21–23].

## Treatments

### TIRS

TIRS will be achieved using foam sclerotherapy. This involves ultrasound assessment of the ulcerated area and identification of veins which allow reflux into the ulcer itself. Under ultrasound guidance, a small needle is used to inject a sclerosant foam directly into those veins identified for treatment. The sclerosant Sotradecol (sodium-tetradecyl-sulphate) will be used for all patients in the trial. The liquid form of Sotradecol will be made into foam by agitating 1-ml Sotradecol with 4 ml air in two 5-ml syringes in a modified Tessari method [24].

### Axial ablation

All patients randomised to AA will have mechanical occlusion with chemical assistance (MOCA). Because this technique is non-thermal, it can be used in more superficial veins, facilitating use in a wider range of anatomies than a thermal technique. Clarivein™ (Vascular Insights LLC, Quincy, USA) is a device consisting of a rotating wire within a catheter. The catheter allows the infusion of sclerosant, while the rapid rotation of the wire scores the intima of the vein, generating an inflammatory response. The infusion of the sclerosant via the same catheter as the wire ensures that it is spread around the full circumference of the vein by the wire as it rotates, further augmenting the inflammatory response, causing occlusion of the vein initially, followed by obliteration. Sotradecol will also be used as the sclerosant in these patients.

## Aim

The aim of this trial is to answer the following research question.

In adult patients with venous ulcers, are TIRS and AA equivalent in their ability to increase the proportion of ulcers that heal completely within 6 months of treatment?

Null hypothesis: there is a clinically significant difference in ulcer healing.

Alternative hypothesis: there is no clinically significant difference in ulcer healing.

## Objectives

### Primary objective

The primary objective is to determine if the proportion of ulcers healing within 6 months of treatment with AA of varicose veins by MOCA or TIRS by peri-ulcer foam sclerotherapy is equivalent.

### Secondary objective

The secondary objective is to determine if there is a significant difference between the two treatments in:

1. Absolute reduction in ulcer size
2. Relative reduction in ulcer size
3. Time to ulcer healing, in those patients whose ulcer has fully healed by the end of the trial
4. Wound regeneration
5. Objective venous disease severity
6. Patient-assessed quality of life

## Methods

### Statement of trial design

This is a prospective, assessor-blinded randomised controlled trial. The primary trial centre will be Leg Ulcer Centre Ireland operating in Roscommon University Hospital, with oversight from the Department of Vascular Surgery, University Hospital Galway (UHG). Other centres may come on board in the future.

### Randomisation

Once enrolled, patients will be stratified by ulcer size (< 5 cm^2^, 5.1 to 10 cm^2^, 10.1 to 25 cm^2^, > 25 cm^2^) and will be randomised to either arm on a 1:1 basis.

#### Sequence generation

Randomisation will be done using a computer-generated random number sequence, stratified by ulcer size.

#### Allocation concealment

Randomisation will be performed using sealed opaque envelopes containing treatment allocation, sequentially marked within each stratification level, to be opened by the operating surgeon immediately before intervention.

#### Implementation

On enrolment the principal investigator will assign a trial number to the patient and be informed of the ulcer’s size. An envelope will then be taken from the appropriate stratum. The trial number will be written on the envelope, and the envelope passed to the surgical team. The team performing the procedure will sign the envelope sealed, and open it in the operating room immediately before the procedure. A member of the surgical team will then take appropriate consent from the patient for the specific procedure being carried out.

### Sample size

Power calculation [25] was informed by existing evidence [11–13, 15], predominantly the EVRA trial [15]. EVRA showed a 95% success rate for ulcer healing at 24 weeks. We anticipated a more modest success rate because our exclusion criteria did not limit participants to those with new ulcers. In addition, TIRS has very limited existing evidence with reporting healing ranging from 83 to 100% [21, 23]. An 80% success rate was felt to be reasonable for both treatments in a non-inferiority trial. A cohort of 308 patients (154 per arm) was required to ensure that if the null hypothesis should be rejected, the limits of a two-sided 90% confidence interval would exclude a difference between the groups of more than 15%, with 90% power at 5% significance level.

Given that existing evidence for TIRS consists mainly of small case series with widely varying healing rates, we planned once the first 50 cases exited the trial (either healed or completed 6 months of follow-up) as a pilot to inform a further power calculation for both superiority and non-inferiority outcomes.

### Outcomes

#### Primary outcome

The primary outcome is the proportion of ulcers which have healed by the time they are reviewed at 24 weeks.

#### Secondary outcomes

The following are the secondary outcomes:

1. Time to ulcer healing, in those ulcers where complete healing is achieved
2. Absolute reduction in ulcer size
3. Relative reduction in ulcer size as a percentage of original ulcer area
4. Wound regeneration as indicated by changes in Bates-Jensen Wound Assessment Tool (BWAT) score over time
5. Change in Venous Clinical Severity Score (VCSS) monthly from randomisation to exit from the study
6. Changes in Charing Cross Venous Ulcer Quesionnaire (CCVUQ) score from randomisation to exit from the study

The BWAT is a tool originally derived for assessing pressure ulcers [26], which has been validated for venous ulcer use [27].

The VCSS is an objective measure of the severity of venous disease, encompassing both ulceration and wound factors, as well as non-wound related signs and symptoms [28].

The CCVUQ is a quality of life questionnaire specific to venous leg ulcers [29].

### Safety

#### Definition of an adverse event

Any medical misadventure encountered as a direct or indirect result of treatment received as part of the trial.

#### Definition of a serious adverse event

Any adverse event, as defined, that results in hospitalisation or prolonged hospital stay, permanent or prolonged incapacity, significant disruption to daily life, threat to life, death, or any congenital abnormality arising from treatment, will be deemed a serious adverse event.

#### Anticipated adverse events

The procedures involved in this trial are typically safe and well tolerated. Though any serious adverse event is unlikely, there are known potential adverse events with both treatments. These include the following:

1. Pain
2. Bleeding
3. Infection
4. Phlebitis
5. Staining of skin
6. Palpable subcutaneous lumps
7. New ulceration
8. Deep vein thrombosis
9. Pulmonary embolism, including life-threatening or fatal embolism
10. Stroke
11. Nerve injury leading to pain/paraesthesia/reduced sensation
12. Skin breakdown or hypersensitivity reaction secondary to compression/dressings
13. Adverse or hypersensitivity reaction to local anaesthetic or Sotradecol

#### Adverse event reporting

The principal investigator should be notified of all adverse events. In the event of multiple similar adverse events, or any serious adverse event, the trial monitoring committee and local ethics committee will be notified, and risk analysis is performed.

### Trial treatment

Patients enrolling in the trial will treated with either TIRS or AA according to their randomly assigned group and will then be placed in compression. Compression will take the form of two- or four-layer compression bandaging, using either Profore™ (Smith & Nephew, London, UK) or Coban™ (3M, Maplewood, MN, USA).

### Trial monitoring

The principal investigator will take responsibility for the day-to-day management of the trial supervised by the supervising investigator. Meetings will be held every 2 weeks between the principal investigator and supervising investigator to monitor recruitment, data collection and problems as they arise. The trial monitoring committee will meet as necessary to review safety issues. An independent data monitoring committee has not been established as the trial interventions are low-risk and consist of already-available and recognised treatment strategies. Any safety or equipoise concerns will be reported to those members of the Department of Vascular Surgery in University Hospital Galway who are not involved in the conduct of the trial for their consideration. This will include the results of the interim analysis.

### Personnel

The principal investigator will manage this trial, along with the help from the on-site tissue viability nurse and the co-authors. The principal investigator will specifically have the responsibility for screening and enrolment of patients, data collection at randomisation and follow-up, and statistical analysis.

The trial nurse(s) will assess all wounds prior to allocation and perform blinded assessment at each subsequent follow-up visit.

### Method of blinding

The trial will be assessor blinded. The trial nurse(s) will assess all wounds prior to randomisation and will be blinded to treatment allocation throughout, performing assessments at each follow-up visit. Patients will be instructed not to reveal the nature of their treatment in follow-up. The operation note from the intervention will be filed in the patient notes to allow intentional or emergency access. All members of the trial team involved in data collection and analysis will not be present when the envelope is opened at the time of treatment.

Any treatment decisions required beyond the trial protocol will be made by a member of the surgical team who is already unblinded.

Ultrasound assessment of treated veins will be avoided unless otherwise indicated, to minimise risk of inadvertent unblinding of assessors.

### Ethical approval

Ethical approval has been obtained through Galway University Hospitals Clinical Research Ethics Committee, the Research Ethics Committee governing Roscommon University Hospital. Approval was granted on 18 June 2020, ref. no. C.A. 2416, before recruitment commenced.

### Screening of patients

Patients will be recruited from Leg Ulcer Centre Ireland, a dedicated leg ulcer clinic in Roscommon University Hospital, drawing referrals from the entire Saolta Hospital Group in the West of Ireland. General practices in the area will receive written information in advance of the trial commencing to encourage direct referral to the clinic. All patients attending this clinic with a leg ulcer will be screened for eligibility including an ultrasound assessment by a member of the surgical team. Written trial information will be provided to each eligible patient, and the option to defer enrolment for a period of consideration will be offered.

### Inclusion criteria

Patients will be eligible for the trial provided that the following criteria are met:

- Primary or recurrent venous leg ulcer
- Long or short saphenous vein reflux confirmed on ultrasound assessment, defined as retrograde flow lasting for > 0.5 s in the standing position
- Ankle-Brachial pressure Index (ABI) ≥ 0.8 (if ulceration prevents ABI, Toe-Brachial Index (TBI) ≥ 0.5 acceptable) or a palpable pulse
- Ulcer size between 1 and 200 cm^2^
- Patient suitable for full compression bandaging

### Exclusion criteria

Patients will be excluded if any of the following apply:

1. Pregnancy (or breastfeeding and needing to feed within 48 h of treatment).
2. Active infection of ulcer, or infection within the last 2 weeks.
3. Leg ulcer of non-venous aetiology as determined by clinical assessment.
4. Isolated perforator vein reflux only.
5. Evidence of deep venous insufficiency or thrombosis.
6. Known hypersensitivity to Sotradecol or similar sclerosants.
7. Previous inability to tolerate compression bandages.
8. Presence of any contraindications for the use of compression bandages:
  - Absence of a palpable pulse, and Ankle Brachial Index (ABI) < 0.8
  - Decompensated congestive cardiac failure (NYHA class IV)
  - Known hypersensitivity to any of the component materials
9. Patients unable to provide informed consent.
10. Patients attending the leg ulcer clinic already will be excluded from enrolment with the same ulcer but will be eligible to enrol with a contralateral ulcer. Recurrent ipsilateral ulcers will not be excluded.

### Consent

Each patient who meets the eligibility criteria will receive a full explanation of the trial aims and rationale, as well as the concept of equipoise. To participate, each patient will have to provide written informed consent to participation, treatment, and data processing. Risks and benefits of both trial treatments will be explained, along with potential complications of compression therapy. At this point they will be given the opportunity to enrol, take time to consider, or not enrol with the assurance that non-participation will not adversely impact further treatment, nor will leaving the trial during follow-up. Three copies of the consent form will be signed (one each for the patient, the patient’s hospital notes and the trial database). Consent for data storage will be discussed and permission sought to contact patients in the future to seek further consent for any further use of collected data.

### Data collection

After screening and enrolment, clinical and demographic data will be collected for each participant using a trial case report form (CRF). These data will be entered into the trial database on an on-going basis.

#### Data protection/storage

The trial database will be stored on a password-protected, desktop computer in UHG. A backup copy will be prepared at the end of each data entry session and stored in a locked filing cabinet. The original data entry CRF will be retained with a copy of the consent form in a locked trial filing cabinet within the Vascular Research Unit. All data shall be monitored by approved trial personnel only and processed and stored in the strictest confidence in accordance with Irish and European Union data protection law. All data will be retained in the care of the principal investigator for a period of 5 years from the closure of the trial.

#### Baseline characteristics

After screening, enrolment and provision of informed consent, the principal investigator or trial nurse will collect the following demographic data:

1. Age
2. Sex
3. Height
4. Weight
5. Body mass index (BMI)
6. Diabetes
7. Smoking
8. Anaemia
9. ABI (or TBI if ulceration prevents cuff application at the ankle)
10. Immunosuppression
11. Medications at time of randomisation and treatment

#### Ulcer characteristics

Along with the above baseline characteristics, the following data related to the wound itself will be documented:

1. Ulcer location
2. Ulcer duration
3. Ulcer area (in cm^2^)
4. Depth (in terms of tissue layers involved)
5. Condition of wound edges
6. Undermining
7. Necrotic tissue type
8. Necrotic tissue amount (as a percentage of wound area)
9. Exudate type
10. Exudate amount
11. Surrounding skin colour
12. Surrounding tissue oedema
13. Surrounding tissue induration
14. Granulation (as a percentage of wound area)
15. Epithelialisation (as a percentage of wound area)
16. Pain (present or not, and relieved by medications or not)

At follow-up visits only:

17. Compliance with compression

These data will be recorded by the trial nurse and a BWAT score and VCSS will be calculated.

At each review appointment, the same data will be collected, and progress of the wound assessed using the BWAT. VCSS will also be monitored monthly.

#### Quality of life data

At their initial visit, and when exiting the trial (ulcer healed or completion of 6-month follow-up), participants will complete a CCVUQ questionnaire [29].

### Treatment regimen

Once patients have been assigned a trial number and randomised, their sealed allocation envelope will be retained. Where possible, enrolment, allocation and treatment will occur on the same day to maximise adherence to trial treatments.

The interventions being tested involve the following core steps

#### TIRS

I. Ultrasound guided puncture of the desired vein using a butterfly needle
II. Injection of up to 10 ml Sotradecol foam (max 2 ml 1% Sotradecol agitated with 8 ml air to give 10 ml foam) under ultrasound guidance
III. Massage of the foam as needed into desired vessels

#### AA

I. Ultrasound guided micropuncture of the desired axial vein
II. Placement of a 5 French-gauge sheath
III. Passage of the Clarivein™ device to the proximal end of the treatment area
IV. Activation of the device, followed by withdrawal at a rate of 1 mm/s, while simultaneously infusing Sotradecol.

Variations of these treatments are at the discretion of the treating surgeon as long as the above steps are met. After treatment, all patients will be placed in compression bandages. They will then be followed up monthly at the same site with changes of their compression bandages in between by their local public health nurse (PHN) weekly, or more frequently as needed. If at any follow-up visit, their ulcer is seen to have healed, the time at which it healed will be noted in weeks from intervention. At this point, they will have completed their follow-up and will exit the trial. Patients in whom the ulcer has not fully healed at 24 weeks will also exit the trial. In either case subsequent treatment will be at the discretion of the surgical team.

### Statistical analysis

Statistical analysis will be performed using StatsDirect Statistical Package version 3 (StatsDirect Ltd., Cambridge, UK.). All analyses will be performed on an intention-to-treat basis, but a per-protocol analysis will also be performed. Continuous variables will be presented as mean (± standard deviation) or median (± interquartile range) based on distribution, with 95% confidence intervals. They will be analysed using Student’s *T*-test for parametric and Mann-Whitney *U* test for non-parametric data. Categorical variables will be described in absolute numbers with percentage frequencies. The chi-squared and Fisher’s exact test will be used.

Trial allocation will be concealed during interim statistical analysis unless a significant difference is found at this point which warrants stopping of the trial or the interim analysis indicates the trial could not be completed within a reasonable timeframe.

### Criteria for discontinuation

Should reasonable justification for termination or suspension of the trial be encountered the principal investigator will notify the regional research ethics committee and all investigators involved in the trial in writing.

- Unacceptable risk to participants due to unexpected significant adverse events
- Interim demonstration of a significant difference in efficacy
- Interim power calculation indicating that significantly more participants would be required, which would prevent the trial being completed within a reasonable timeframe
- Intolerable discomfort during treatment or compression, or inability to adhere to protocol for any other reason leading to insufficient compliance with randomisation
- Incomplete data collection precluding coherent analysis

### Protocol violations

Inability to tolerate AA or TIRS, or compression bandages will constitute a violation of protocol. Failure to attend follow-up on a single occasion will not constitute a protocol violation, and patients will be contacted by phone to arrange further follow-up. All analyses will be performed primarily on an intention-to-treat basis, but a per-protocol analysis will also be performed.

#### Losses to follow-up

At enrolment, all patients will be informed of the importance of attending follow-up according to the trial protocol. The principal investigator will endeavour to contact any patients missing a follow-up appointment.

#### Drop-out criteria

Participants will be considered to have left the study if, despite all efforts of the trial team, no reliable determination can be made regarding the primary outcome.

### Schedule

#### Referral

Once referred for ulcer management, patients will be seen within 6 weeks, with the aim of keeping all waiting periods under 1 month.

#### First clinic visit

At their 1st visit to the clinic, patients will be screened, and if found to be eligible, the principal investigator or another designated trial investigator will discuss enrolment. If willing, patients will have their baseline data and wound assessment recorded as above and be randomised and treated on the same day as screening unless they wish to defer enrolment.

#### Deferred enrolment

Patients wishing to take time to think about enrolment will be offered the chance and brought back to the clinic at an interval agreeable to them.

Patients already attending the ulcer clinic will be eligible for enrolment if they present with a new contralateral ulcer.

#### Subsequent follow-up

Patients will attend monthly follow-up, with instructions to the PHN changing their dressings to continue compression until follow-up in all cases. At follow-up appointments, patients will fill a VCSS questionnaire, and the trial nurse will assess their wound and document the BWAT score. Non-healed ulcers will be placed in compression with any other dressings or adjuncts as appropriate and continue follow-up.

#### Final follow-up

Once a patient has attended for follow-up and their ulcer has healed, they will exit the trial. If the ulcer does not heal by 24 weeks post-intervention, these patients will exit the trial at this point. At their final visit, patients will have a BWAT score, VCSS and CCVUQ questionnaire recorded. All patients completing follow-up, either through completion of 6 months or having healed their ulcer, will be seen by a member of the clinical team in the ulcer clinic who will determine ongoing follow-up management.

### Dissemination

#### Registration

The trial is registered at ClinicalTrials.gov, reference number NCT04484168.

#### Reporting

The trial protocol will be published in a suitable peer-reviewed journal and recorded in keeping with the SPIRIT guidelines [30]. Trial results will be submitted for peer-reviewed publication regardless of outcome. The principal investigator will have the responsibility for drafting the manuscript, and all co-authors will have editorial input.

## Discussion

TIRS has not yet been tested in a randomised trial, so this will be the first rigorous test of the efficacy of TIRS. By comparing TIRS against AA, there is the opportunity for significant savings in healthcare resources because TIRS using sclerosant foam is less expensive and less time consuming than AA, as well as less invasive. If the results of TIRS are comparable to those of AA, then a significant savings resources could be made.

The EVRA trial required that all participants undergo treatment to the lowest point of reflux but did not specify that all participants undergo formal ablation, and patients may have been treated with foam from the lowest point of reflux, in a manner similar to the aim of TIRS. The results of this trial will help to refine best practice in how reflux ablation to aid in ulcer healing is delivered in practice. The results of this trial may also serve to support the findings of EVRA if similar high healing rates are seen with either or both forms of ablation.

## Trial status

Protocol version 3

AAVTIRS began recruiting on 21 July 2020 and was registered on 23 July 2020.

Enrolment is ongoing. Projected completion of enrolment is in the first half of 2022, with completion is projected in late 2022 follow-up after the final participants have completed 6 months of follow-up.

## Supplementary Information

**Additional file 1: Appendix 1.** Patient consent form. **Appendix 2.** Reporting checklist for protocol of a clinical trial.

## Data Availability

The datasets during and/or analysed during the current study are available from the corresponding author on reasonable request.

## Abbreviations

AA: Axial Ablation
TIRS: Terminal interruption of the reflux source
BWAT: Bates-Jensen Wound Assessment Tool
CCVUQ: Charing Cross Venous Ulcer Questionnaire
VCSS: Venous Clinical Severity Score
MOCA: Mechanical Occlusion with Chemical Assistance
CRF: Case report form
BMI: Body mass index
ABI: Ankle Brachial Index
UHG: University Hospital Galway
PHN: Public Health Nurse
